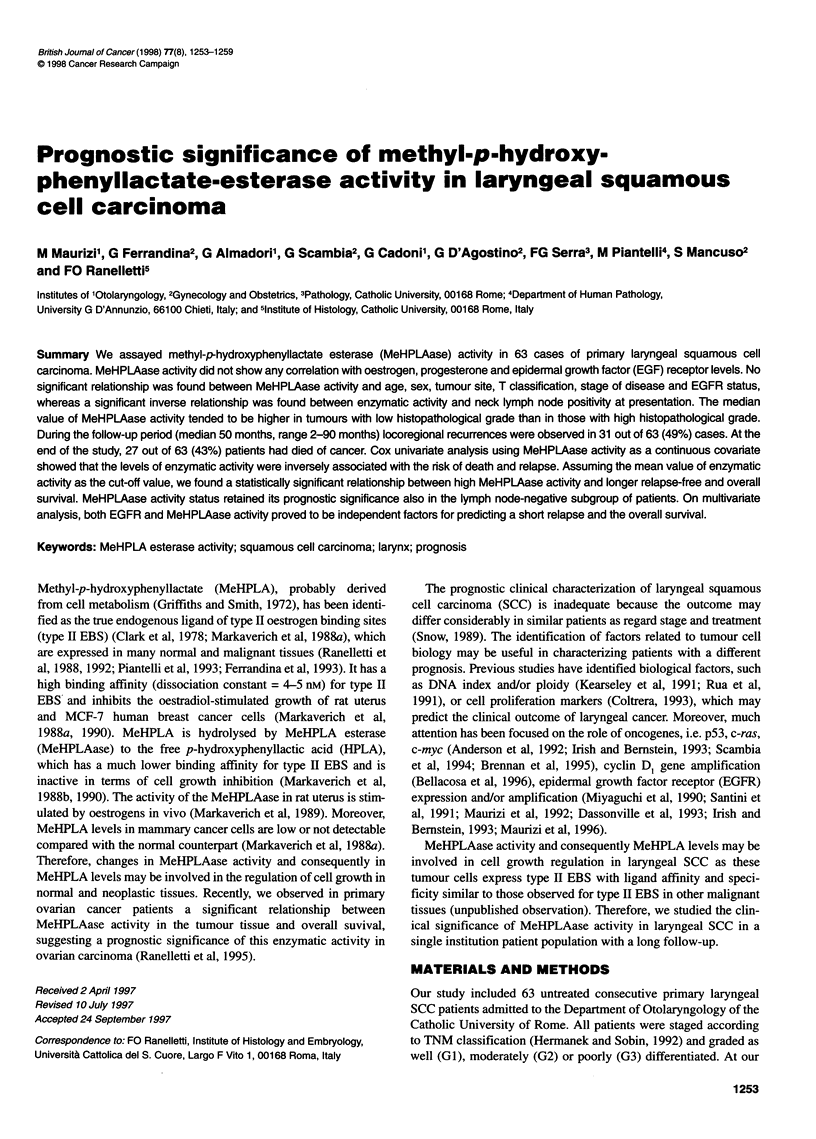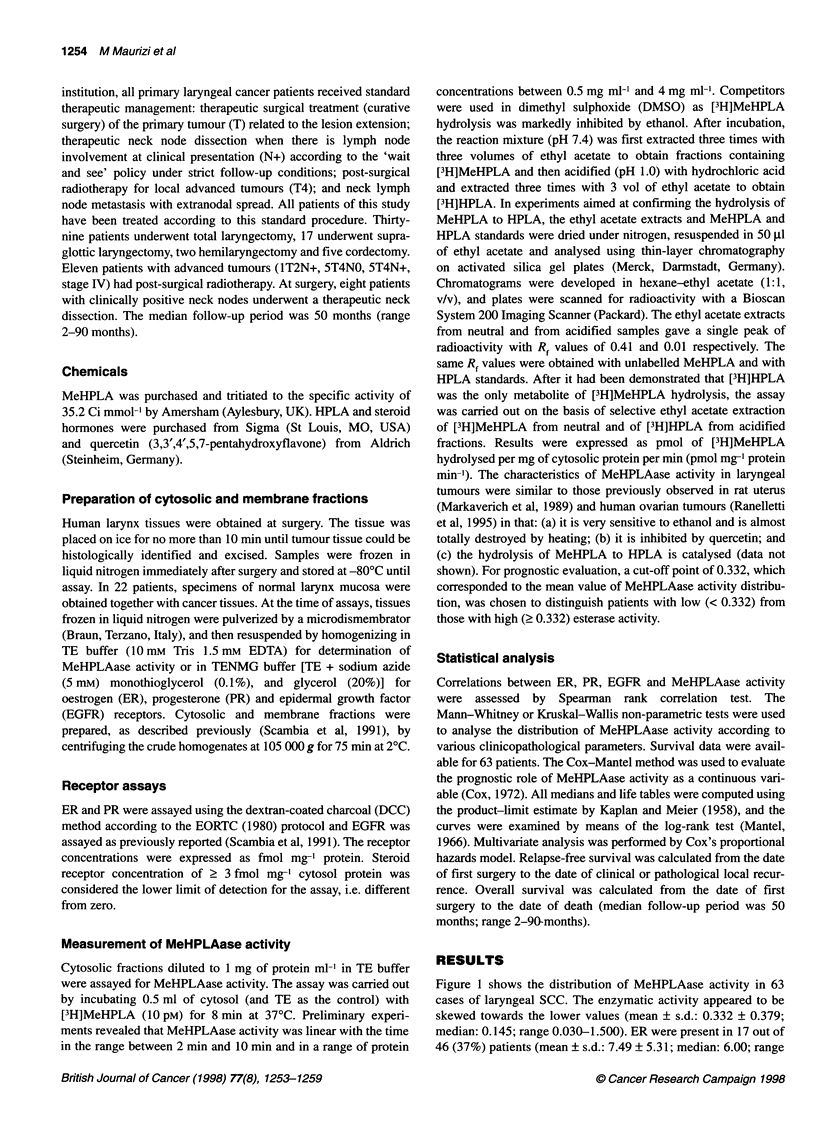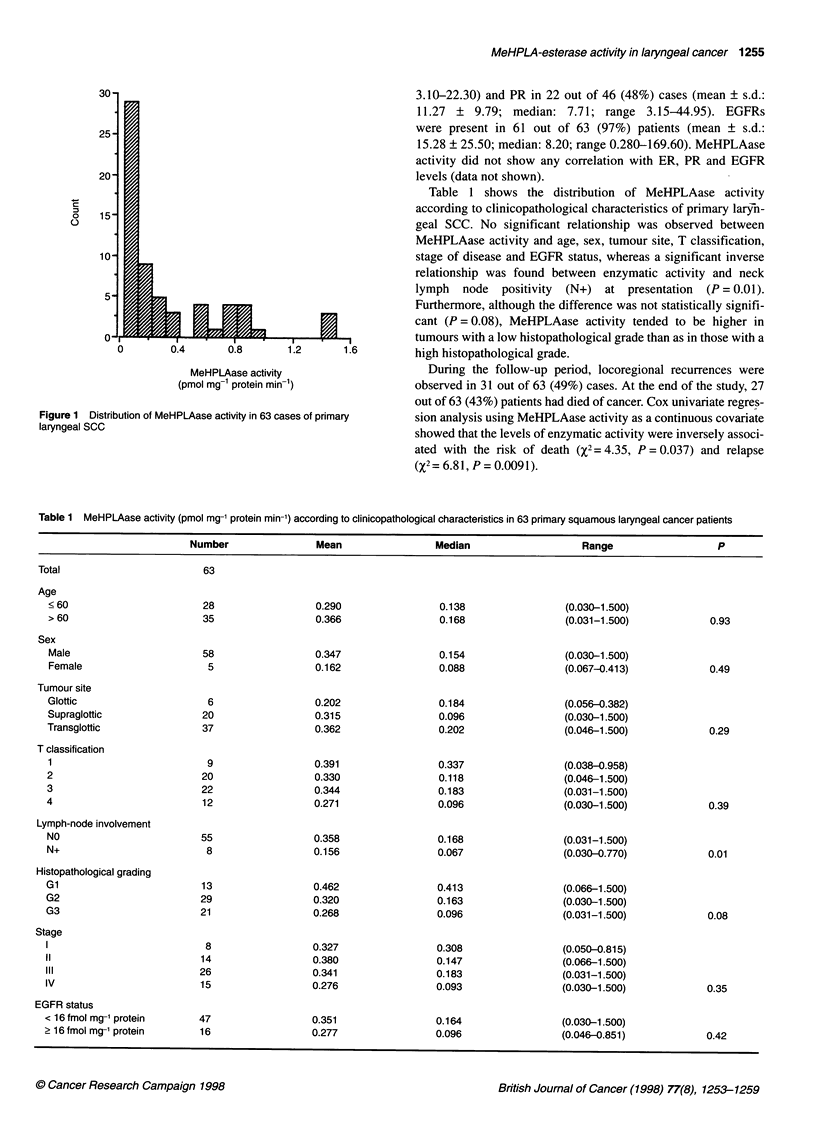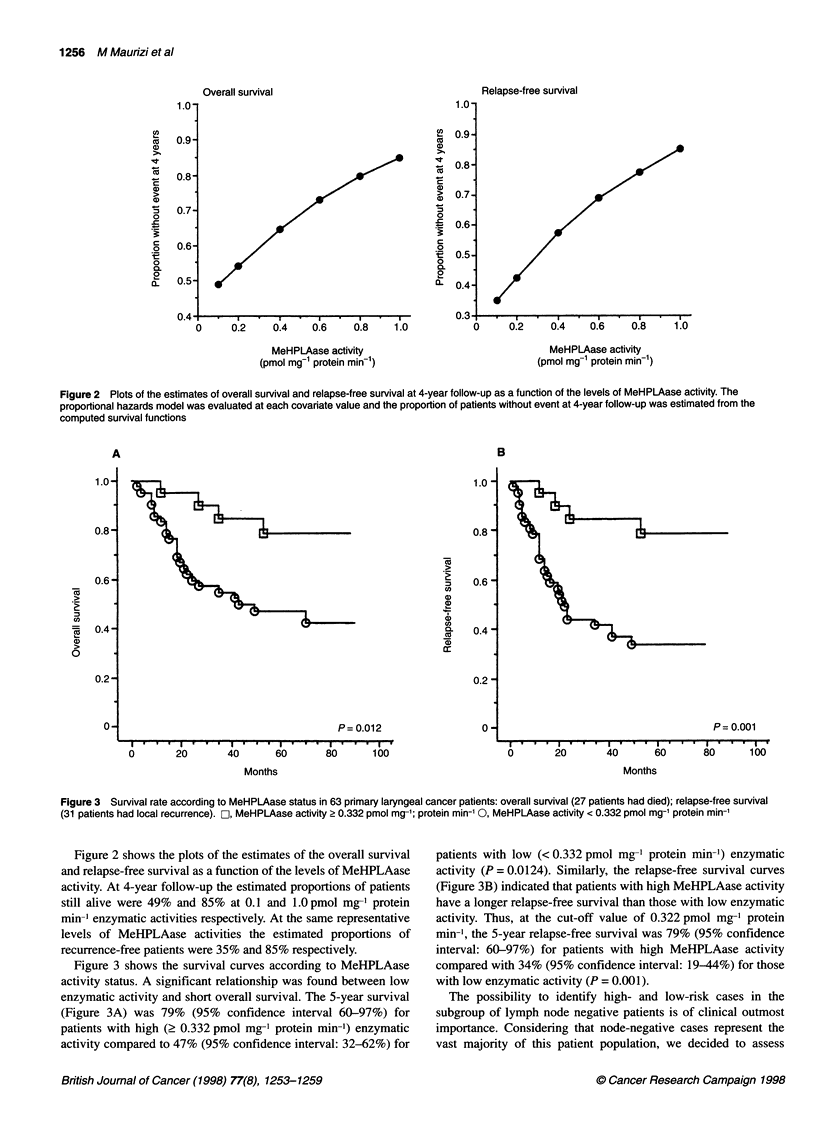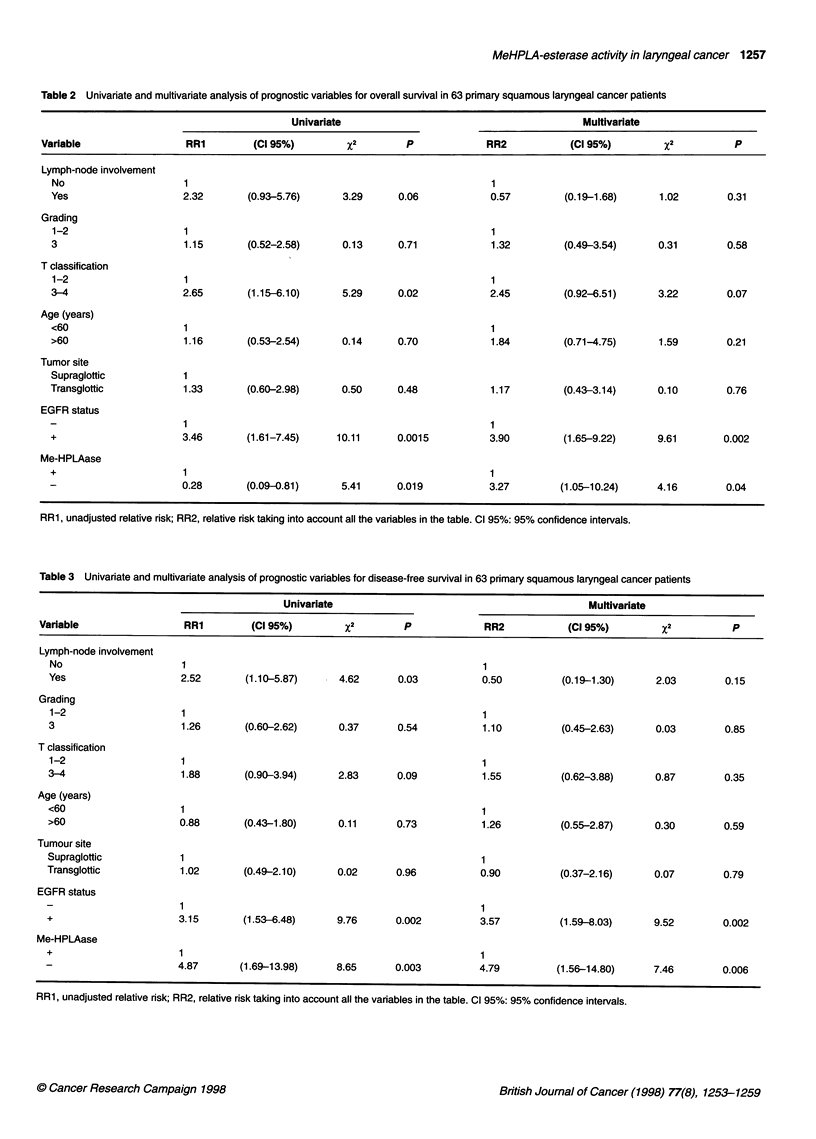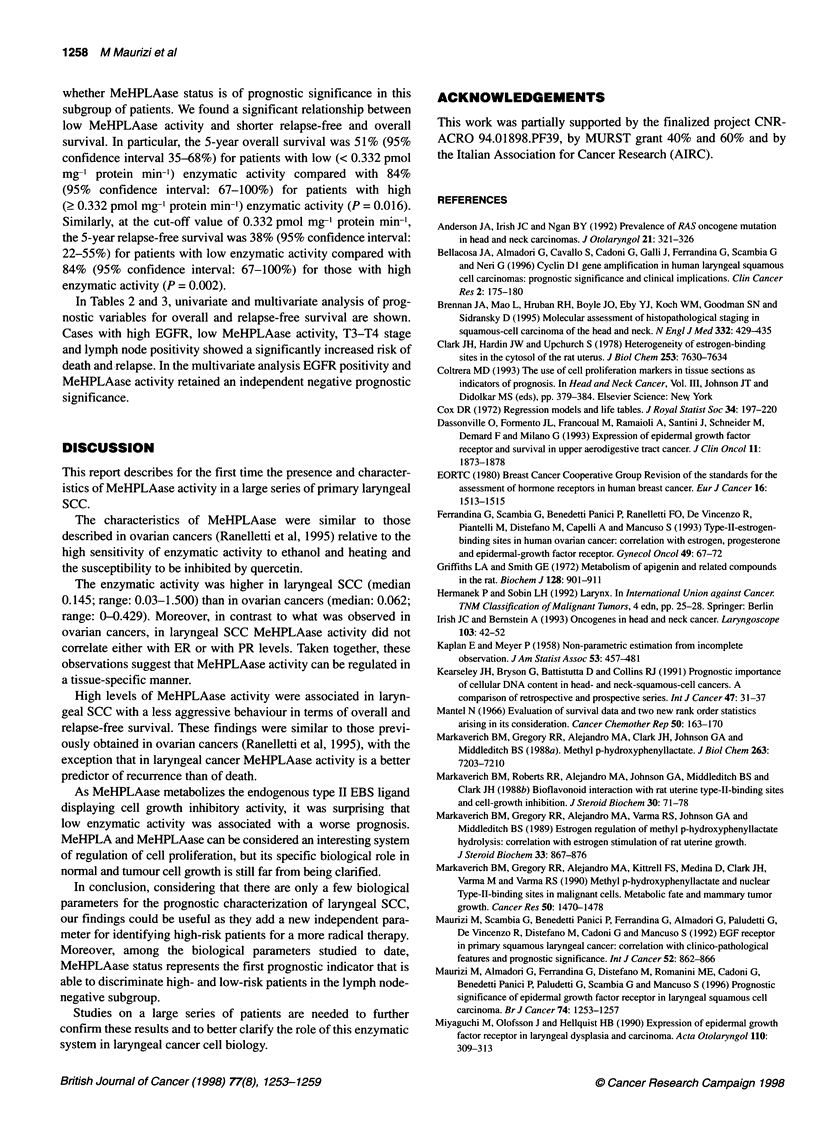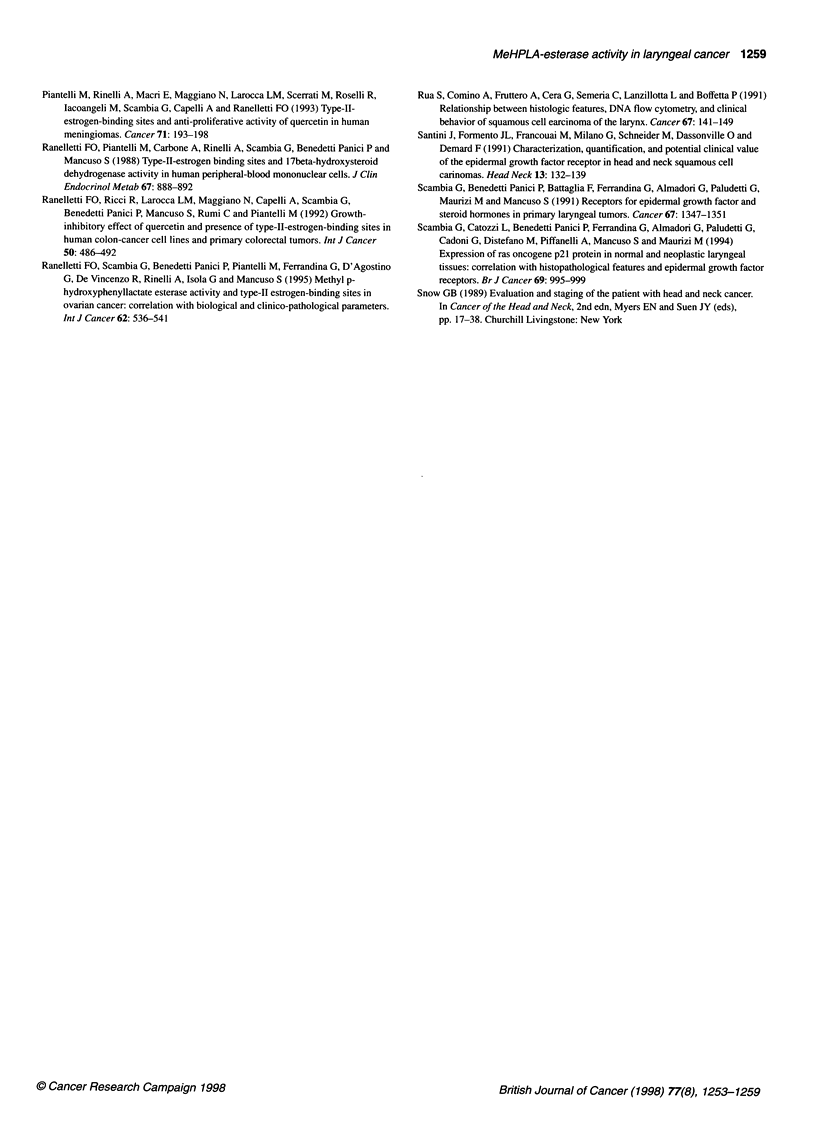# Prognostic significance of methyl-p-hydroxy-phenyllactate-esterase activity in laryngeal squamous cell carcinoma.

**DOI:** 10.1038/bjc.1998.210

**Published:** 1998-04

**Authors:** M. Maurizi, G. Ferrandina, G. Almadori, G. Scambia, G. Cadoni, G. D'Agostino, F. G. Serra, M. Piantelli, S. Mancuso, F. O. Ranelletti

**Affiliations:** Institute of Otolaryngology, Catholic University, Rome, Italy.

## Abstract

We assayed methyl-p-hydroxyphenyllactate esterase (MeHPLAase) activity in 63 cases of primary laryngeal squamous cell carcinoma. MeHPLAase activity did not show any correlation with oestrogen, progesterone and epidermal growth factor (EGF) receptor levels. No significant relationship was found between MeHPLAase activity and age, sex, tumour site, T classification, stage of disease and EGFR status, whereas a significant inverse relationship was found between enzymatic activity and neck lymph node positivity at presentation. The median value of MeHPLAase activity tended to be higher in tumours with low histopathological grade than in those with high histopathological grade. During the follow-up period (median 50 months, range 2-90 months) locoregional recurrences were observed in 31 out of 63 (49%) cases. At the end of the study, 27 out of 63 (43%) patients had died of cancer. Cox univariate analysis using MeHPLAase activity as a continuous covariate showed that the levels of enzymatic activity were inversely associated with the risk of death and relapse. Assuming the mean value of enzymatic activity as the cut-off value, we found a statistically significant relationship between high MeHPLAase activity and longer relapse-free and overall survival. MeHPLAase activity status retained its prognostic significance also in the lymph node-negative subgroup of patients. On multivariate analysis, both EGFR and MeHPLAase activity proved to be independent factors for predicting a short relapse and the overall survival.


British Journal of Cancer (1998) 77(8), 1253–1259
© 1998 Cancer Research Campaign

### Prognostic significance of methyl-*p*-hydroxyphenyllactate-esterase activity in laryngeal squamous cell carcinoma

**M Maurizi[1], G Ferrandina[2], G Almadori[1], G Scambia[2], G Cadoni[1], G D'Agostino[2], FG Serra[3], M Piantelli[4], S Mancuso[2] and FO Ranelletti[5]**

Institutes of [1]Otolaryngology, [2]Gynecology and Obstetrics, [3]Pathology, Catholic University, 00168 Rome; [4]Department of Human Pathology, University G D'Annunzio, 66100 Chieti, Italy; and [5]Institute of Histology, Catholic University, 00168 Rome, Italy

**Summary** We assayed methyl-*p*-hydroxyphenyllactate esterase (MeHPLAase) activity in 63 cases of primary laryngeal squamous cell carcinoma. MeHPLAase activity did not show any correlation with oestrogen, progesterone and epidermal growth factor (EGF) receptor levels. No significant relationship was found between MeHPLAase activity and age, sex, tumour site, T classification, stage of disease and EGFR status, whereas a significant inverse relationship was found between enzymatic activity and neck lymph node positivity at presentation. The median value of MeHPLAase activity tended to be higher in tumours with low histopathological grade than in those with high histopathological grade. During the follow-up period (median 50 months, range 2–90 months) locoregional recurrences were observed in 31 out of 63 (49%) cases. At the end of the study, 27 out of 63 (43%) patients had died of cancer. Cox univariate analysis using MeHPLAase activity as a continuous covariate showed that the levels of enzymatic activity were inversely associated with the risk of death and relapse. Assuming the mean value of enzymatic activity as the cut-off value, we found a statistically significant relationship between high MeHPLAase activity and longer relapse-free and overall survival. MeHPLAase activity status retained its prognostic significance also in the lymph node-negative subgroup of patients. On multivariate analysis, both EGFR and MeHPLAase activity proved to be independent factors for predicting a short relapse and the overall survival.

**Keywords:** MeHPLA esterase activity; squamous cell carcinoma; larynx; prognosis

Methyl-*p*-hydroxyphenyllactate (MeHPLA), probably derived from cell metabolism (Griffiths and Smith, 1972), has been identified as the true endogenous ligand of type II oestrogen binding sites (type II EBS) (Clark et al, 1978; Markaverich et al, 1988*a*), which are expressed in many normal and malignant tissues (Ranelletti et al, 1988, 1992; Piantelli et al, 1993; Ferrandina et al, 1993). It has a high binding affinity (dissociation constant = 4–5 nM) for type II EBS and inhibits the oestradiol-stimulated growth of rat uterus and MCF-7 human breast cancer cells (Markaverich et al, 1988*a*, 1990). MeHPLA is hydrolysed by MeHPLA esterase (MeHPLAase) to the free *p*-hydroxyphenyllactic acid (HPLA), which has a much lower binding affinity for type II EBS and is inactive in terms of cell growth inhibition (Markaverich et al, 1988b, 1990). The activity of the MeHPLAase in rat uterus is stimulated by oestrogens in vivo (Markaverich et al, 1989). Moreover, MeHPLA levels in mammary cancer cells are low or not detectable compared with the normal counterpart (Markaverich et al, 1988*a*). Therefore, changes in MeHPLAase activity and consequently in MeHPLA levels may be involved in the regulation of cell growth in normal and neoplastic tissues. Recently, we observed in primary ovarian cancer patients a significant relationship between MeHPLAase activity in the tumour tissue and overall suvival, suggesting a prognostic significance of this enzymatic activity in ovarian carcinoma (Ranelletti et al, 1995).

*Received 2 April 1997*
*Revised 10 July 1997*
*Accepted 24 September 1997*

*Correspondence to:* FO Ranelletti, Institute of Histology and Embryology, Università Cattolica del S. Cuore, Largo F Vito 1, 00168 Roma, Italy

The prognostic clinical characterization of laryngeal squamous cell carcinoma (SCC) is inadequate because the outcome may differ considerably in similar patients as regard stage and treatment (Snow, 1989). The identification of factors related to tumour cell biology may be useful in characterizing patients with a different prognosis. Previous studies have identified biological factors, such as DNA index and/or ploidy (Kearseley et al, 1991; Rua et al, 1991), or cell proliferation markers (Coltrera, 1993), which may predict the clinical outcome of laryngeal cancer. Moreover, much attention has been focused on the role of oncogenes, i.e. p53, c-*ras*, c-*myc* (Anderson et al, 1992; Irish and Bernstein, 1993; Scambia et al, 1994; Brennan et al, 1995), cyclin $D_1$ gene amplification (Bellacosa et al, 1996), epidermal growth factor receptor (EGFR) expression and/or amplification (Miyaguchi et al, 1990; Santini et al, 1991; Maurizi et al, 1992; Dassonville et al, 1993; Irish and Bernstein, 1993; Maurizi et al, 1996).

MeHPLAase activity and consequently MeHPLA levels may be involved in cell growth regulation in laryngeal SCC as these tumour cells express type II EBS with ligand affinity and specificity similar to those observed for type II EBS in other malignant tissues (unpublished observation). Therefore, we studied the clinical significance of MeHPLAase activity in laryngeal SCC in a single institution patient population with a long follow-up.

### MATERIALS AND METHODS

Our study included 63 untreated consecutive primary laryngeal SCC patients admitted to the Department of Otolaryngology of the Catholic University of Rome. All patients were staged according to TNM classification (Hermanek and Sobin, 1992) and graded as well (G1), moderately (G2) or poorly (G3) differentiated. At our institution, all primary laryngeal cancer patients received standard therapeutic management: therapeutic surgical treatment (curative surgery) of the primary tumour (T) related to the lesion extension; therapeutic neck node dissection when there is lymph node involvement at clinical presentation (N+) according to the 'wait and see' policy under strict follow-up conditions; post-surgical radiotherapy for local advanced tumours (T4); and neck lymph node metastasis with extranodal spread. All patients of this study have been treated according to this standard procedure. Thirty-nine patients underwent total laryngectomy, 17 underwent supraglottic laryngectomy, two hemilaryngectomy and five cordectomy. Eleven patients with advanced tumours (1T2N+, 5T4N0, 5T4N+, stage IV) had post-surgical radiotherapy. At surgery, eight patients with clinically positive neck nodes underwent a therapeutic neck dissection. The median follow-up period was 50 months (range 2–90 months).

### Chemicals

MeHPLA was purchased and tritiated to the specific activity of 35.2 Ci $mmol^{-1}$ by Amersham (Aylesbury, UK). HPLA and steroid hormones were purchased from Sigma (St Louis, MO, USA) and quercetin (3,3′,4′,5,7-pentahydroxyflavone) from Aldrich (Steinheim, Germany).

### Preparation of cytosolic and membrane fractions

Human larynx tissues were obtained at surgery. The tissue was placed on ice for no more than 10 min until tumour tissue could be histologically identified and excised. Samples were frozen in liquid nitrogen immediately after surgery and stored at –80°C until assay. In 22 patients, specimens of normal larynx mucosa were obtained together with cancer tissues. At the time of assays, tissues frozen in liquid nitrogen were pulverized by a microdismembrator (Braun, Terzano, Italy), and then resuspended by homogenizing in TE buffer (10 mM Tris 1.5 mM EDTA) for determination of MeHPLAase activity or in TENMG buffer [TE + sodium azide (5 mM) monothioglycerol (0.1%), and glycerol (20%)] for oestrogen (ER), progesterone (PR) and epidermal growth factor (EGFR) receptors. Cytosolic and membrane fractions were prepared, as described previously (Scambia et al, 1991), by centrifuging the crude homogenates at 105 000 ***g*** for 75 min at 2°C.

### Receptor assays

ER and PR were assayed using the dextran-coated charcoal (DCC) method according to the EORTC (1980) protocol and EGFR was assayed as previously reported (Scambia et al, 1991). The receptor concentrations were expressed as fmol $mg^{-1}$ protein. Steroid receptor concentration of ≥ 3 fmol $mg^{-1}$ cytosol protein was considered the lower limit of detection for the assay, i.e. different from zero.

### Measurement of MeHPLAase activity

Cytosolic fractions diluted to 1 mg of protein $ml^{-1}$ in TE buffer were assayed for MeHPLAase activity. The assay was carried out by incubating 0.5 ml of cytosol (and TE as the control) with [$^3$H]MeHPLA (10 pM) for 8 min at 37°C. Preliminary experiments revealed that MeHPLAase activity was linear with the time in the range between 2 min and 10 min and in a range of protein concentrations between 0.5 mg $ml^{-1}$ and 4 mg $ml^{-1}$. Competitors were used in dimethyl sulphoxide (DMSO) as [$^3$H]MeHPLA hydrolysis was markedly inhibited by ethanol. After incubation, the reaction mixture (pH 7.4) was first extracted three times with three volumes of ethyl acetate to obtain fractions containing [$^3$H]MeHPLA and then acidified (pH 1.0) with hydrochloric acid and extracted three times with 3 vol of ethyl acetate to obtain [$^3$H]HPLA. In experiments aimed at confirming the hydrolysis of MeHPLA to HPLA, the ethyl acetate extracts and MeHPLA and HPLA standards were dried under nitrogen, resuspended in 50 µl of ethyl acetate and analysed using thin-layer chromatography on activated silica gel plates (Merck, Darmstadt, Germany). Chromatograms were developed in hexane–ethyl acetate (1:1, v/v), and plates were scanned for radioactivity with a Bioscan System 200 Imaging Scanner (Packard). The ethyl acetate extracts from neutral and from acidified samples gave a single peak of radioactivity with $R_f$ values of 0.41 and 0.01 respectively. The same $R_f$ values were obtained with unlabelled MeHPLA and with HPLA standards. After it had been demonstrated that [$^3$H]HPLA was the only metabolite of [$^3$H]MeHPLA hydrolysis, the assay was carried out on the basis of selective ethyl acetate extraction of [$^3$H]MeHPLA from neutral and of [$^3$H]HPLA from acidified fractions. Results were expressed as pmol of [$^3$H]MeHPLA hydrolysed per mg of cytosolic protein per min (pmol $mg^{-1}$ protein $min^{-1}$). The characteristics of MeHPLAase activity in laryngeal tumours were similar to those previously observed in rat uterus (Markaverich et al, 1989) and human ovarian tumours (Ranelletti et al, 1995) in that: (a) it is very sensitive to ethanol and is almost totally destroyed by heating; (b) it is inhibited by quercetin; and (c) the hydrolysis of MeHPLA to HPLA is catalysed (data not shown). For prognostic evaluation, a cut-off point of 0.332, which corresponded to the mean value of MeHPLAase activity distribution, was chosen to distinguish patients with low (< 0.332) from those with high (≥ 0.332) esterase activity.

### Statistical analysis

Correlations between ER, PR, EGFR and MeHPLAase activity were assessed by Spearman rank correlation test. The Mann–Whitney or Kruskal–Wallis non-parametric tests were used to analyse the distribution of MeHPLAase activity according to various clinicopathological parameters. Survival data were available for 63 patients. The Cox–Mantel method was used to evaluate the prognostic role of MeHPLAase activity as a continuous variable (Cox, 1972). All medians and life tables were computed using the product–limit estimate by Kaplan and Meier (1958), and the curves were examined by means of the log-rank test (Mantel, 1966). Multivariate analysis was performed by Cox's proportional hazards model. Relapse-free survival was calculated from the date of first surgery to the date of clinical or pathological local recurrence. Overall survival was calculated from the date of first surgery to the date of death (median follow-up period was 50 months; range 2–90-months).

### RESULTS

Figure 1 shows the distribution of MeHPLAase activity in 63 cases of laryngeal SCC. The enzymatic activity appeared to be skewed towards the lower values (mean ± s.d.: 0.332 ± 0.379; median: 0.145; range 0.030–1.500). ER were present in 17 out of 46 (37%) patients (mean ± s.d.: 7.49 ± 5.31; median: 6.00; range

© Cancer Research Campaign 1998

**Figure 1** Distribution of MeHPLAase activity in 63 cases of primary laryngeal SCC

3.10–22.30) and PR in 22 out of 46 (48%) cases (mean ± s.d.: 11.27 ± 9.79; median: 7.71; range 3.15–44.95). EGFRs were present in 61 out of 63 (97%) patients (mean ± s.d.: 15.28 ± 25.50; median: 8.20; range 0.280–169.60). MeHPLAase activity did not show any correlation with ER, PR and EGFR levels (data not shown).

Table 1 shows the distribution of MeHPLAase activity according to clinicopathological characteristics of primary laryngeal SCC. No significant relationship was observed between MeHPLAase activity and age, sex, tumour site, T classification, stage of disease and EGFR status, whereas a significant inverse relationship was found between enzymatic activity and neck lymph node positivity (N+) at presentation ($P = 0.01$). Furthermore, although the difference was not statistically significant ($P = 0.08$), MeHPLAase activity tended to be higher in tumours with a low histopathological grade than as in those with a high histopathological grade.

During the follow-up period, locoregional recurrences were observed in 31 out of 63 (49%) cases. At the end of the study, 27 out of 63 (43%) patients had died of cancer. Cox univariate regression analysis using MeHPLAase activity as a continuous covariate showed that the levels of enzymatic activity were inversely associated with the risk of death ($\chi^2 = 4.35$, $P = 0.037$) and relapse ($\chi^2 = 6.81$, $P = 0.0091$).

**Table 1** MeHPLAase activity (pmol mg$^{-1}$ protein min$^{-1}$) according to clinicopathological characteristics in 63 primary squamous laryngeal cancer patients

| | Number | Mean | Median | Range | *P* |
|---|---|---|---|---|---|
| Total | 63 | | | | |
| Age | | | | | |
| ≤ 60 | 28 | 0.290 | 0.138 | (0.030–1.500) | |
| > 60 | 35 | 0.366 | 0.168 | (0.031–1.500) | 0.93 |
| Sex | | | | | |
| Male | 58 | 0.347 | 0.154 | (0.030–1.500) | |
| Female | 5 | 0.162 | 0.088 | (0.067–0.413) | 0.49 |
| Tumour site | | | | | |
| Glottic | 6 | 0.202 | 0.184 | (0.056–0.382) | |
| Supraglottic | 20 | 0.315 | 0.096 | (0.030–1.500) | |
| Transglottic | 37 | 0.362 | 0.202 | (0.046–1.500) | 0.29 |
| T classification | | | | | |
| 1 | 9 | 0.391 | 0.337 | (0.038–0.958) | |
| 2 | 20 | 0.330 | 0.118 | (0.046–1.500) | |
| 3 | 22 | 0.344 | 0.183 | (0.031–1.500) | |
| 4 | 12 | 0.271 | 0.096 | (0.030–1.500) | 0.39 |
| Lymph-node involvement | | | | | |
| N0 | 55 | 0.358 | 0.168 | (0.031–1.500) | |
| N+ | 8 | 0.156 | 0.067 | (0.030–0.770) | 0.01 |
| Histopathological grading | | | | | |
| G1 | 13 | 0.462 | 0.413 | (0.066–1.500) | |
| G2 | 29 | 0.320 | 0.163 | (0.030–1.500) | |
| G3 | 21 | 0.268 | 0.096 | (0.031–1.500) | 0.08 |
| Stage | | | | | |
| I | 8 | 0.327 | 0.308 | (0.050–0.815) | |
| II | 14 | 0.380 | 0.147 | (0.066–1.500) | |
| III | 26 | 0.341 | 0.183 | (0.031–1.500) | |
| IV | 15 | 0.276 | 0.093 | (0.030–1.500) | 0.35 |
| EGFR status | | | | | |
| < 16 fmol mg$^{-1}$ protein | 47 | 0.351 | 0.164 | (0.030–1.500) | |
| ≥ 16 fmol mg$^{-1}$ protein | 16 | 0.277 | 0.096 | (0.046–0.851) | 0.42 |

© Cancer Research Campaign 1998

**Figure 2** Plots of the estimates of overall survival and relapse-free survival at 4-year follow-up as a function of the levels of MeHPLAase activity. The proportional hazards model was evaluated at each covariate value and the proportion of patients without event at 4-year follow-up was estimated from the computed survival functions

**Figure 3** Survival rate according to MeHPLAase status in 63 primary laryngeal cancer patients: overall survival (27 patients had died); relapse-free survival (31 patients had local recurrence). □, MeHPLAase activity ≥ 0.332 pmol mg$^{-1}$; protein min$^{-1}$ ○, MeHPLAase activity < 0.332 pmol mg$^{-1}$ protein min$^{-1}$

Figure 2 shows the plots of the estimates of the overall survival and relapse-free survival as a function of the levels of MeHPLAase activity. At 4-year follow-up the estimated proportions of patients still alive were 49% and 85% at 0.1 and 1.0 pmol mg$^{-1}$ protein min$^{-1}$ enzymatic activities respectively. At the same representative levels of MeHPLAase activities the estimated proportions of recurrence-free patients were 35% and 85% respectively.

Figure 3 shows the survival curves according to MeHPLAase activity status. A significant relationship was found between low enzymatic activity and short overall survival. The 5-year survival (Figure 3A) was 79% (95% confidence interval 60–97%) for patients with high (≥ 0.332 pmol mg$^{-1}$ protein min$^{-1}$) enzymatic activity compared to 47% (95% confidence interval: 32–62%) for patients with low (< 0.332 pmol mg$^{-1}$ protein min$^{-1}$) enzymatic activity ($P = 0.0124$). Similarly, the relapse-free survival curves (Figure 3B) indicated that patients with high MeHPLAase activity have a longer relapse-free survival than those with low enzymatic activity. Thus, at the cut-off value of 0.322 pmol mg$^{-1}$ protein min$^{-1}$, the 5-year relapse-free survival was 79% (95% confidence interval: 60–97%) for patients with high MeHPLAase activity compared with 34% (95% confidence interval: 19–44%) for those with low enzymatic activity ($P = 0.001$).

The possibility to identify high- and low-risk cases in the subgroup of lymph node negative patients is of clinical outmost importance. Considering that node-negative cases represent the vast majority of this patient population, we decided to assess

© Cancer Research Campaign 1998

**Table 2** Univariate and multivariate analysis of prognostic variables for overall survival in 63 primary squamous laryngeal cancer patients

| | Univariate | | | | Multivariate | | | |
|---|---|---|---|---|---|---|---|---|
| **Variable** | **RR1** | **(CI 95%)** | $\chi^2$ | ***P*** | **RR2** | **(CI 95%)** | $\chi^2$ | ***P*** |
| Lymph-node involvement | | | | | | | | |
| No | 1 | | | | 1 | | | |
| Yes | 2.32 | (0.93–5.76) | 3.29 | 0.06 | 0.57 | (0.19–1.68) | 1.02 | 0.31 |
| Grading | | | | | | | | |
| 1–2 | 1 | | | | 1 | | | |
| 3 | 1.15 | (0.52–2.58) | 0.13 | 0.71 | 1.32 | (0.49–3.54) | 0.31 | 0.58 |
| T classification | | | | | | | | |
| 1–2 | 1 | | | | 1 | | | |
| 3–4 | 2.65 | (1.15–6.10) | 5.29 | 0.02 | 2.45 | (0.92–6.51) | 3.22 | 0.07 |
| Age (years) | | | | | | | | |
| <60 | 1 | | | | 1 | | | |
| >60 | 1.16 | (0.53–2.54) | 0.14 | 0.70 | 1.84 | (0.71–4.75) | 1.59 | 0.21 |
| Tumor site | | | | | | | | |
| Supraglottic | 1 | | | | | | | |
| Transglottic | 1.33 | (0.60–2.98) | 0.50 | 0.48 | 1.17 | (0.43–3.14) | 0.10 | 0.76 |
| EGFR status | | | | | | | | |
| – | 1 | | | | 1 | | | |
| + | 3.46 | (1.61–7.45) | 10.11 | 0.0015 | 3.90 | (1.65–9.22) | 9.61 | 0.002 |
| Me-HPLAase | | | | | | | | |
| + | 1 | | | | 1 | | | |
| – | 0.28 | (0.09–0.81) | 5.41 | 0.019 | 3.27 | (1.05–10.24) | 4.16 | 0.04 |

RR1, unadjusted relative risk; RR2, relative risk taking into account all the variables in the table. CI 95%: 95% confidence intervals.

**Table 3** Univariate and multivariate analysis of prognostic variables for disease-free survival in 63 primary squamous laryngeal cancer patients

| | Univariate | | | | Multivariate | | | |
|---|---|---|---|---|---|---|---|---|
| **Variable** | **RR1** | **(CI 95%)** | $\chi^2$ | ***P*** | **RR2** | **(CI 95%)** | $\chi^2$ | ***P*** |
| Lymph-node involvement | | | | | | | | |
| No | 1 | | | | 1 | | | |
| Yes | 2.52 | (1.10–5.87) | 4.62 | 0.03 | 0.50 | (0.19–1.30) | 2.03 | 0.15 |
| Grading | | | | | | | | |
| 1–2 | 1 | | | | 1 | | | |
| 3 | 1.26 | (0.60–2.62) | 0.37 | 0.54 | 1.10 | (0.45–2.63) | 0.03 | 0.85 |
| T classification | | | | | | | | |
| 1–2 | 1 | | | | 1 | | | |
| 3–4 | 1.88 | (0.90–3.94) | 2.83 | 0.09 | 1.55 | (0.62–3.88) | 0.87 | 0.35 |
| Age (years) | | | | | | | | |
| <60 | 1 | | | | 1 | | | |
| >60 | 0.88 | (0.43–1.80) | 0.11 | 0.73 | 1.26 | (0.55–2.87) | 0.30 | 0.59 |
| Tumour site | | | | | | | | |
| Supraglottic | 1 | | | | 1 | | | |
| Transglottic | 1.02 | (0.49–2.10) | 0.02 | 0.96 | 0.90 | (0.37–2.16) | 0.07 | 0.79 |
| EGFR status | | | | | | | | |
| – | 1 | | | | 1 | | | |
| + | 3.15 | (1.53–6.48) | 9.76 | 0.002 | 3.57 | (1.59–8.03) | 9.52 | 0.002 |
| Me-HPLAase | | | | | | | | |
| + | 1 | | | | 1 | | | |
| – | 4.87 | (1.69–13.98) | 8.65 | 0.003 | 4.79 | (1.56–14.80) | 7.46 | 0.006 |

RR1, unadjusted relative risk; RR2, relative risk taking into account all the variables in the table. CI 95%: 95% confidence intervals.

© Cancer Research Campaign 1998

whether MeHPLAase status is of prognostic significance in this subgroup of patients. We found a significant relationship between low MeHPLAase activity and shorter relapse-free and overall survival. In particular, the 5-year overall survival was 51% (95% confidence interval 35–68%) for patients with low (< 0.332 pmol $mg^{-1}$ protein $min^{-1}$) enzymatic activity compared with 84% (95% confidence interval: 67–100%) for patients with high (≥ 0.332 pmol $mg^{-1}$ protein $min^{-1}$) enzymatic activity ($P = 0.016$). Similarly, at the cut-off value of 0.332 pmol $mg^{-1}$ protein $min^{-1}$, the 5-year relapse-free survival was 38% (95% confidence interval: 22–55%) for patients with low enzymatic activity compared with 84% (95% confidence interval: 67–100%) for those with high enzymatic activity ($P = 0.002$).

In Tables 2 and 3, univariate and multivariate analysis of prognostic variables for overall and relapse-free survival are shown. Cases with high EGFR, low MeHPLAase activity, T3–T4 stage and lymph node positivity showed a significantly increased risk of death and relapse. In the multivariate analysis EGFR positivity and MeHPLAase activity retained an independent negative prognostic significance.

### DISCUSSION

This report describes for the first time the presence and characteristics of MeHPLAase activity in a large series of primary laryngeal SCC.

The characteristics of MeHPLAase were similar to those described in ovarian cancers (Ranelletti et al, 1995) relative to the high sensitivity of enzymatic activity to ethanol and heating and the susceptibility to be inhibited by quercetin.

The enzymatic activity was higher in laryngeal SCC (median 0.145; range: 0.03–1.500) than in ovarian cancers (median: 0.062; range: 0–0.429). Moreover, in contrast to what was observed in ovarian cancers, in laryngeal SCC MeHPLAase activity did not correlate either with ER or with PR levels. Taken together, these observations suggest that MeHPLAase activity can be regulated in a tissue-specific manner.

High levels of MeHPLAase activity were associated in laryngeal SCC with a less aggressive behaviour in terms of overall and relapse-free survival. These findings were similar to those previously obtained in ovarian cancers (Ranelletti et al, 1995), with the exception that in laryngeal cancer MeHPLAase activity is a better predictor of recurrence than of death.

As MeHPLAase metabolizes the endogenous type II EBS ligand displaying cell growth inhibitory activity, it was surprising that low enzymatic activity was associated with a worse prognosis. MeHPLA and MeHPLAase can be considered an interesting system of regulation of cell proliferation, but its specific biological role in normal and tumour cell growth is still far from being clarified.

In conclusion, considering that there are only a few biological parameters for the prognostic characterization of laryngeal SCC, our findings could be useful as they add a new independent parameter for identifying high-risk patients for a more radical therapy. Moreover, among the biological parameters studied to date, MeHPLAase status represents the first prognostic indicator that is able to discriminate high- and low-risk patients in the lymph node-negative subgroup.

Studies on a large series of patients are needed to further confirm these results and to better clarify the role of this enzymatic system in laryngeal cancer cell biology.

### ACKNOWLEDGEMENTS

This work was partially supported by the finalized project CNR-ACRO 94.01898.PF39, by MURST grant 40% and 60% and by the Italian Association for Cancer Research (AIRC).

### REFERENCES

Anderson JA, Irish JC and Ngan BY (1992) Prevalence of *RAS* oncogene mutation in head and neck carcinomas. *J Otolaryngol* **21**: 321–326

Bellacosa JA, Almadori G, Cavallo S, Cadoni G, Galli J, Ferrandina G, Scambia G and Neri G (1996) Cyclin D1 gene amplification in human laryngeal squamous cell carcinomas: prognostic significance and clinical implications. *Clin Cancer Res* **2**: 175–180

Brennan JA, Mao L, Hruban RH, Boyle JO, Eby YJ, Koch WM, Goodman SN and Sidransky D (1995) Molecular assessment of histopathological staging in squamous-cell carcinoma of the head and neck. *N Engl J Med* **332**: 429–435

Clark JH, Hardin JW and Upchurch S (1978) Heterogeneity of estrogen-binding sites in the cytosol of the rat uterus. *J Biol Chem* **253**: 7630–7634

Coltrera MD (1993) The use of cell proliferation markers in tissue sections as indicators of prognosis. In *Head and Neck Cancer*, Vol. III, Johnson JT and Didolkar MS (eds), pp. 379–384. Elsevier Science: New York

Cox DR (1972) Regression models and life tables. *J Royal Statist Soc* **34**: 197–220

Dassonville O, Formento JL, Francoual M, Ramaioli A, Santini J, Schneider M, Demard F and Milano G (1993) Expression of epidermal growth factor receptor and survival in upper aerodigestive tract cancer. *J Clin Oncol* **11**: 1873–1878

EORTC (1980) Breast Cancer Cooperative Group Revision of the standards for the assessment of hormone receptors in human breast cancer. *Eur J Cancer* **16**: 1513–1515

Ferrandina G, Scambia G, Benedetti Panici P, Ranelletti FO, De Vincenzo R, Piantelli M, Distefano M, Capelli A and Mancuso S (1993) Type-II-estrogen-binding sites in human ovarian cancer: correlation with estrogen, progesterone and epidermal-growth factor receptor. *Gynecol Oncol* **49**: 67–72

Griffiths LA and Smith GE (1972) Metabolism of apigenin and related compounds in the rat. *Biochem J* **128**: 901–911

Hermanek P and Sobin LH (1992) Larynx. In *International Union against Cancer. TNM Classification of Malignant Tumors*, 4 edn, pp. 25–28. Springer: Berlin

Irish JC and Bernstein A (1993) Oncogenes in head and neck cancer. *Laryngoscope* **103**: 42–52

Kaplan E and Meyer P (1958) Non-parametric estimation from incomplete observation. *J Am Statist Assoc* **53**: 457–481

Kearseley JH, Bryson G, Battistutta D and Collins RJ (1991) Prognostic importance of cellular DNA content in head- and neck-squamous-cell cancers. A comparison of retrospective and prospective series. *Int J Cancer* **47**: 31–37

Mantel N (1966) Evaluation of survival data and two new rank order statistics arising in its consideration. *Cancer Chemother Rep* **50**: 163–170

Markaverich BM, Gregory RR, Alejandro MA, Clark JH, Johnson GA and Middleditch BS (1988*a*). Methyl p-hydroxyphenyllactate. *J Biol Chem* **263**: 7203–7210

Markaverich BM, Roberts RR, Alejandro MA, Johnson GA, Middleditch BS and Clark JH (1988*b*) Bioflavonoid interaction with rat uterine type-II-binding sites and cell-growth inhibition. *J Steroid Biochem* **30**: 71–78

Markaverich BM, Gregory RR, Alejandro MA, Varma RS, Johnson GA and Middleditch BS (1989) Estrogen regulation of methyl p-hydroxyphenyllactate hydrolysis: correlation with estrogen stimulation of rat uterine growth. *J Steroid Biochem* **33**: 867–876

Markaverich BM, Gregory RR, Alejandro MA, Kittrell FS, Medina D, Clark JH, Varma M and Varma RS (1990) Methyl p-hydroxyphenyllactate and nuclear Type-II-binding sites in malignant cells. Metabolic fate and mammary tumor growth. *Cancer Res* **50**: 1470–1478

Maurizi M, Scambia G, Benedetti Panici P, Ferrandina G, Almadori G, Paludetti G, De Vincenzo R, Distefano M, Cadoni G and Mancuso S (1992) EGF receptor in primary squamous laryngeal cancer: correlation with clinico-pathological features and prognostic significance. *Int J Cancer* **52**: 862–866

Maurizi M, Almadori G, Ferrandina G, Distefano M, Romanini ME, Cadoni G, Benedetti Panici P, Paludetti G, Scambia G and Mancuso S (1996) Prognostic significance of epidermal growth factor receptor in laryngeal squamous cell carcinoma. *Br J Cancer* **74**: 1253–1257

Miyaguchi M, Olofsson J and Hellquist HB (1990) Expression of epidermal growth factor receptor in laryngeal dysplasia and carcinoma. *Acta Otolaryngol* **110**: 309–313

© Cancer Research Campaign 1998

Piantelli M, Rinelli A, Macri E, Maggiano N, Larocca LM, Scerrati M, Roselli R, Iacoangeli M, Scambia G, Capelli A and Ranelletti FO (1993) Type-II-estrogen-binding sites and anti-proliferative activity of quercetin in human meningiomas. *Cancer* **71**: 193–198

Ranelletti FO, Piantelli M, Carbone A, Rinelli A, Scambia G, Benedetti Panici P and Mancuso S (1988) Type-II-estrogen binding sites and 17beta-hydroxysteroid dehydrogenase activity in human peripheral-blood mononuclear cells. *J Clin Endocrinol Metab* **67**: 888–892

Ranelletti FO, Ricci R, Larocca LM, Maggiano N, Capelli A, Scambia G, Benedetti Panici P, Mancuso S, Rumi C and Piantelli M (1992) Growth-inhibitory effect of quercetin and presence of type-II-estrogen-binding sites in human colon-cancer cell lines and primary colorectal tumors. *Int J Cancer* **50**: 486–492

Ranelletti FO, Scambia G, Benedetti Panici P, Piantelli M, Ferrandina G, D'Agostino G, De Vincenzo R, Rinelli A, Isola G and Mancuso S (1995) Methyl p-hydroxyphenyllactate esterase activity and type-II estrogen-binding sites in ovarian cancer: correlation with biological and clinico-pathological parameters. *Int J Cancer* **62**: 536–541

Rua S, Comino A, Fruttero A, Cera G, Semeria C, Lanzillotta L and Boffetta P (1991) Relationship between histologic features, DNA flow cytometry, and clinical behavior of squamous cell earcinoma of the larynx. *Cancer* **67**: 141–149

Santini J, Formento JL, Francouai M, Milano G, Schneider M, Dassonville O and Demard F (1991) Characterization, quantification, and potential clinical value of the epidermal growth factor receptor in head and neck squamous cell carinomas. *Head Neck* **13**: 132–139

Scambia G, Benedetti Panici P, Battaglia F, Ferrandina G, Almadori G, Paludetti G, Maurizi M and Mancuso S (1991) Receptors for epidermal growth factor and steroid hormones in primary laryngeal tumors. *Cancer* **67**: 1347–1351

Scambia G, Catozzi L, Benedetti Panici P, Ferrandina G, Almadori G, Paludetti G, Cadoni G, Distefano M, Piffanelli A, Mancuso S and Maurizi M (1994) Expression of ras oncogene p21 protein in normal and neoplastic laryngeal tissues: correlation with histopathological features and epidermal growth factor receptors. *Br J Cancer* **69**: 995–999

Snow GB (1989) Evaluation and staging of the patient with head and neck cancer. In *Cancer of the Head and Neck*, 2nd edn, Myers EN and Suen JY (eds), pp. 17–38. Churchill Livingstone: New York

© Cancer Research Campaign 1998